# Pancreatic Macrophages: Critical Players in Obesity-Promoted Pancreatic Cancer

**DOI:** 10.3390/cancers12071946

**Published:** 2020-07-17

**Authors:** Yaroslav Teper, Guido Eibl

**Affiliations:** Department of Surgery, David Geffen School of Medicine at UCLA, University of California at Los Angeles, Los Angeles, CA 90095, USA; YTeper@mednet.ucla.edu

**Keywords:** obesity, pancreatic cancer, macrophages, inflammation, acinar-to-ductal metaplasia

## Abstract

Obesity is a known risk factor for the development of pancreatic cancer, one of the deadliest types of malignancies. In recent years it has become clear that the pancreatic microenvironment is critically involved and a contributing factor in accelerating pancreatic neoplasia. In this context obesity-associated chronic inflammation plays an important role. Among several immune cells, macrophages have been shown to contribute to obesity-induced tissue inflammation. This review article summarizes the current knowledge about the role of pancreatic macrophages in early pancreatic cancer development. It describes the heterogenous origin and mixture of pancreatic macrophages, their role in pancreatic endocrine and exocrine pathology, and the impact of obesity on islet and stromal macrophages. A model is postulated, by which during obesity monocytes are recruited into the pancreas, where they are polarized into pro-inflammatory macrophages that drive early pancreatic neoplasia. This occurs in the presence of local inflammatory, metabolic, and endocrine signals. A stronger appreciation and more detailed knowledge about the role of macrophages in early pancreatic cancer development will lead to innovative preventive or interceptive strategies.

## 1. Introduction

Pancreatic ductal adenocarcinoma (PDAC) is one of the most lethal malignancies with an overall 5-year survival rate of approximately 9% [1]. Current treatment options are mostly surgical resection in localized disease and chemotherapy. However, only a minority of patients are candidates for surgery and the response rates and survival benefits of present chemotherapeutic regimens are disappointingly low. A valid conceptual approach to improve the outcome of pancreatic cancer patients is to intercept early disease progression before the cancer becomes invasive and metastatic. This concept requires the identification of patients who are at high risk of developing the disease and/or detection of early disease as well as a detailed understanding of mechanisms that drive early tumor development.

It is well accepted today that the majority of PDACs arise from precursor lesions, e.g., pancreatic intraepithelial neoplasia (PanIN), which progress from early PanIN-1 to advanced PanIN-3 and eventually invasive PDAC. These PanIN lesions accumulate key genetic alterations, which are also found in PDAC. Essentially all invasive PDACs contain an activating *KRAS* mutation (typically *G12D*), which can already be detected in early PanIN lesions [2]. Recent exome sequencing established *KRAS* to be the most frequently mutated gene in PDAC (~95%) [3,4]. These findings led to the stepwise carcinogenesis paradigm, in which *KRAS* mutations are characterized as initiating events. However, there is also evidence that at least a fraction of human PDACs can develop outside the PanIN-PDAC sequence paradigm [5,6,7]. Several studies have shown that Kras mutations can be detected at higher frequency in pancreatic juice and stool of patients with PDAC compared to normal patients and patients with benign pancreatic pathologies [8,9,10], making this disease potentially amenable to prevention or early interception.

Mouse models have demonstrated that PanINs can develop from metaplastic lesions in the pancreas, termed acinar-to-ductal metaplasia (ADM). ADMs occur frequently in the pancreas, e.g., during pancreatic inflammation, but are usually reversible. However, in the presence of an oncogenic Kras mutation in acinar cells, ADMs may persist and progress to early PanIN lesions [11]. There has been intense interest in elucidating the cell of origin in PDAC development. Several studies have reported that while the presence of oncogenic Kras in pancreatic acinar cells will lead to ADM and PanINs, the expression of oncogenic Kras in pancreatic ductal cells is associated with PDAC development independent of PanIN development via non-mucinous lesions [12,13]. It is increasingly recognized that the pancreatic microenvironment has a profound influence and actually drives early PDAC development. In this context, elegant preclinical studies have shown that pro-inflammatory macrophages play a critical role in ADM formation and early ADM to PanIN transition [14,15].

Large epidemiologic studies and preclinical animal models have clearly established that obesity increases the risk for developing PDAC [16,17,18,19,20,21]. An NIH (National Institutes of Health) analysis revealed that around 16.9% of pancreatic cancer cases may be attributable to being overweight [22]. Considering the high prevalence of obesity worldwide, a detailed understanding of how obesity promotes PDAC progression is of critical importance and would enable the development of interceptive strategies. Several mechanisms have been implicated in how obesity may accelerate cancer development and growth, including chronic inflammation, insulin resistance with hyperinsulinemia, altered adipokine production and sex hormone metabolism, gut dysbiosis, dietary factors, and alteration of the immune response to transformed cells [23,24,25,26,27].

Given the importance of proinflammatory macrophages in pancreatic ADM formation, a concept emerges, in which obesity augments and sustains macrophage-driven pancreatic ADM formation, leading to promotion and acceleration of PDAC development. This review will summarize the current literature and our own data on the effects and mechanisms of obesity on pancreatic macrophages and their potential impact in early PDAC development.

## 2. Obesity and Adipose Tissue Macrophages

Obesity is characterized by a systemic, chronic, low-grade inflammation that is causally linked to the development of insulin resistance. Although the exact molecular signals are still incompletely understood, there is consensus in the scientific literature that macrophage activation in the adipose tissue (AT) contributes to the low-grade, chronic, pro-inflammatory state that is seen in obese subjects. In fact, it has been shown that the expression of inflammatory cytokines, e.g., TNF-α, in AT was almost entirely derived from macrophages [28,29]. In obese AT inflammatory M1-like macrophages are mainly located surrounding dead adipocytes in what is histologically called “crown like structures (CLS)” [30]. During the early phases of obesity development there is an increase in the absolute number of several immune cells in the AT, e.g., neutrophils, NK cells, and macrophages. The percentage of adipose tissue macrophages (ATMs) increases from about 10% of all cells in lean AT to more than 50% in severe obesity in mice [29,31]. The increase of macrophage numbers in obese AT is thereby thought to be mainly caused by the recruitment of Ly6C^+^ CCR2^high^ bone marrow-derived monocytes to the AT through a CCL2 (C-C motif chemokine ligand 2) (MCP-1 (monocyte chemoattractant protein-1))/CCR2 (C-C chemokine receptor type 2)-mediated mechanism, where they differentiate into ATMs [32]. High-fat-diet-fed mice deficient in MCP-1 or CCR2 showed reduced pro-inflammatory M1-like ATMs and displayed markedly decreased AT inflammation and insulin resistance [33,34]. Conversely, over-expression of MCP-1 in AT causes macrophage recruitment in aP2-MCP-1 mice [35].

Lean AT is characterized by M2-like (alternatively activated) macrophages, which maintain an immunosuppressive environment with the presence of Th2-type cytokines, e.g., IL-4, IL-5, and IL-13. During obesity, a phenotypic switch to pro-inflammatory, M1-like (classically activated) macrophages occurs. However, it is now clear that beyond the classical M1/M2 dichotomy in macrophage polarization, inflammatory macrophages in the obese AT often display surface receptor expression distinct from typical M1 polarized macrophages. These ATMs can be activated by a variety of metabolic signals, e.g., free fatty acids (FFA), lipopolysaccharide (LPS), and glucose, and are hence termed metabolically activated macrophages (MMe). For a more detailed description of macrophage recruitment and activation in obese AT the reader is referred to several excellent comprehensive reviews on this topic [36,37,38,39,40,41]. The importance of obesity-induced AT inflammation in PDAC development has been discussed elsewhere [42].

## 3. Obesity and Pancreatic Macrophages

### 3.1. Pancreatic Macrophages under Steady State Conditions

Under steady state conditions a heterogenous population of macrophages of mixed origin exists in the adult pancreas that has a long half-life and is mostly self-maintained [43]. In murine pancreatic islets of Langerhans the only myeloid cells are CD11c, MHCII, F4/80 positive macrophages. Detailed analyses have identified a F4/80^lo^CD11c+ population, which is enriched within the islets (intra-islet macrophages) and F4/80^hi^CD11c– macrophages, which resides in the peripheral islet area (known as peri-islet macrophages) [44]. However, the functional significance of the different islet macrophage populations and their importance for steady state islet physiology is still unclear. The stroma of the exocrine pancreas displayed a complex mixture of leukocytes, including macrophages, dendritic cells, and B and T cells. While islet macrophages show characteristics of M1-like cells, stromal macrophages are mostly tilted towards a M2-like phenotype [43]. Most islet macrophages but not stromal macrophages depend on colony stimulating factor-1 (CSF-1), also known as macrophage-colony stimulating factor (M-CSF), and CSF-1 deficiency was associated with a decreased number of islet macrophages and reduced islet size [43]. Interestingly, under steady state conditions, the number and activation of islet and pancreatic stromal macrophages was independent of CCR2 (C-C chemokine receptor type 2) [43], a major chemokine that is important in the recruitment of blood monocytes into tissues. While both islet and stromal murine macrophages can already be found early after birth (or even during embryologic stages), the number and surface marker expression resembled adult pancreatic macrophages after about 4 weeks [43,45]. The origin of the islet macrophages has been shown to be from adult hematopoietic stem cells (bone marrow derived), while stromal macrophages have a mixed origin from both hematopoietic stem cells and embryonic tissues (yolk sac and fetal liver) [43,46].

### 3.2. Pancreatic Macrophages during Neoplastic Development

The majority of PDACs are thought to arise from preinvasive precursor lesions, e.g., PanINs. These PanINs can develop from transdifferentiating acinar cells, in a process called acinar-to-ductal metaplasia (ADM) [11]. ADMs occur frequently also in acute pancreatitis, but are usually reversible once the initiating trigger subsides, and are thought to represent a pancreatic regeneration program. Animal models and organoid cultures have demonstrated that pro-inflammatory, M1 polarized macrophages can drive ADM during acute pancreatitis, which was mediated through the secretion by macrophages of cytokines, e.g., TNF-α (tumor necrosis factor-alpha) and chemokines, e.g., CCL5/RANTES (regulated upon activation, normal T cell expressed and presumably secreted) [15]. However, in the presence of an oncogenic Kras in pancreatic acinar cells, ADMs may persist and progress to PanINs. It has been shown that acinar cells expressing an oncogenic Kras can attract proinflammatory M1 polarized macrophages, which is mediated by the secretion of soluble intracellular adhesion molecule-1 (ICAM- 1) [14]. Once recruited into the pancreas, these M1 macrophages release proinflammatory cytokines to drive ADM formation and proteinases, e.g., matrix metalloproteinases (MMPs), which help to remodel the pancreatic microenvironment [47,48]. When macrophages in the p48-Cre;LSL-KrasG12D mouse model of PDAC were depleted by treatment with gadolinium (III) chloride, the development and progression of ADM/PanINs was significantly attenuated [14]. In addition, in the same mouse model neutralizing antibodies against ICAM-1 reduced the infiltration of M1 polarized macrophages into the pancreas and attenuated ADM/PanIN formation and progression [14]. The same authors showed in subsequent studies that PanIN (Tuft cell)-derived IL-13 initiates the phenotypic switch from classically activated, proinflammatory M1 polarized macrophages to alternatively activated M2 polarized macrophages [49]. Analyzing human and murine pancreas tissues, M1 macrophages are thereby located mainly around ADMs, while M2 polarized macrophages are predominantly found adjacent to PanIN lesions [49]. A concept emerges, in which proinflammatory M1 macrophages are indispensable for early ADM formation, while M2 macrophages are important in later PanIN/PDAC development. These M2 macrophages (tumor-associated macrophages) are known to contribute to the fibroinflammatory reaction, angiogenesis, and immunosuppression in invasive PDAC [50].

A detailed immune-phenotypic analysis of the pancreas of KrasG12D mice (LSL-KrasG12D/+;Pdx-1-Cre and LSL-KrasG12D/+;p48Cre) revealed that the percentage of CD45+ leukocytes of total cells increased from 7.5% in wildtype mice to 19.5% in PanIN mice (3–10 month old KrasG12D mice harboring only preinvasive PanIN lesions) and almost 50% in PDA mice (KrasG12D mice harboring invasive PDAC) [51]. Of those infiltrating leukocytes the majority were macrophages, which were already present around early PanIN lesions and persisted throughout PDAC development [51]. In another elegant study using the p48-CRE+;LSL-KrasG12D;p53flox/+ (KPC) mouse model, these tumor-associated macrophages were demonstrated to be of heterogeneous origins [52]. In addition to the recruitment of circulating Ly6C^hi^ monocytes, embryologically-derived tissue-resident macrophages are also a major source of tumor-associated macrophages. Using bone marrow transplant and parabiosis studies the authors have shown that Ly6C^hi^ monocytes almost exclusively replenished the MHCII^hi^ subset of tumor-associated macrophages [52]. MHCII (major histocompatibility complex class II) is typically expressed at high levels in proinflammatory M1 macrophages [53]. Interestingly, embryonically-derived tumor-associated macrophages exhibited a pro-fibrotic transcriptional profile and were potent drivers of PDAC progression [52], providing evidence that macrophages with distinct origins have different functional importance during PDAC development and growth.

### 3.3. Pancreatic Islet Macrophages in the Obese State

The impact of obesity on macrophage number and function in the pancreas has mainly been studied in the context of insulin resistance. Obesity is by far the major cause of insulin resistance in humans. Many obese individuals are also prediabetic and may eventually develop type 2 diabetes mellitus (T2DM). In T2DM, macrophage infiltration is increased in pancreatic islets and the number of islet macrophages correlates with the degree of β-cell dysfunction [44,54]. In this context, macrophages in T2DM islets phenotypically are M1-like macrophages. Similarly, human and murine studies have shown that obesity is associated with islet inflammation and increased numbers of islet macrophages [44,55,56]. While islet macrophages during steady state conditions show a low turn-over rate, under obese states the proliferation of islet macrophages is substantially increased [44]. Systemic metabolic disturbances, e.g., hyperglycemia, or islet-derived local factors, e.g., ATP released from stressed β-cells or accumulation of IAPP (islet amyloid polypeptide), can thereby trigger the initial islet inflammation [55,57,58]. The secretion of proinflammatory cytokines, mainly by macrophages, during islet inflammation is thought to lead to β-cell apoptosis and suppressed glucose stimulated insulin secretion (GSIS) [59]. In this context, macrophage-secreted IL-1β seems to be critical, but other factors might play a role as well [55]. High concentrations of IL-1β have been reported to suppress genes important for β-cell differentiation leading to β-cell apoptosis and dedifferentiation into islet endocrine cells that secrete insulin and glucagon, thereby impairing GSIS [60,61]. In addition to soluble factors, direct cell-cell contact between islet macrophages and β-cells, possibly through nanotubes, seems to be an important mechanism as well, by which macrophages can decrease β-cell insulin secretion and which is increased in obesity [44,55].

Obesity is commonly associated with an expansion of pancreatic islets and β-cells, which secrete increased quantities of insulin to compensate for obesity-induced insulin resistance. In addition to their role in β-cell apoptosis and suppressed GSIS, macrophages have been shown to be able to stimulate β-cell proliferation through a platelet-derived growth factor (PDGF)-PDGF receptor-mediated pathway [44,62]. Besides hyperglycemia, elevated levels of saturated fatty acids (SFA), e.g., palmitate, have been postulated to contribute to islet inflammation and β-cell dysfunction and proliferation in the context of obesity and T2DM through a toll-like receptor 4 (TLR4)-mediated mechanism [63,64]. SFAs can bind to TLR4 receptors on islet macrophages and elicit cytokine production, e.g., IL-1β. In addition, SFAs can also activate the TLR4 receptor on β-cells, which then secrete chemokines that can lead to macrophage accumulation in islets [63].

### 3.4. Pancreatic Stromal Macrophages in the Obese State

In contrast to the extensive literature about the important role of islet macrophages in obesity and T2DM, knowledge about the impact of obesity on stromal macrophages in the pancreas is scarce. Our own studies have shown that F4/80+ macrophages are abundant in the pancreas of *LSL-KrasG12D;Pdx-1-Cre* or *LSL-KrasG12D;p48-Cre* mice, known as KC mice, with diet-induced obesity (DIO) [65]. In this model, KC mice fed a diet high in fats and calories (HFCD) displayed a gradual and significant weight gain together with systemic hyperinsulinemia and hyperleptinemia [65,66]. This was associated with an intense fibroinflammatory reaction in the pancreas, as evidenced histologically by robust fibrosis and elevated proinflammatory cytokines, e.g., IL-6 and TNF-α, and acceleration of pancreatic neoplastic development [65,66]. Flow cytometry analyses of immune cells in the pancreas of six-month-old KC mice showed an increase in CD11b^pos^Gr-1^neg^ cells, suggestive of tissue resident macrophages, compared to wildtype pancreas, which was further substantially enhanced by DIO. However, whether DIO initially stimulates the recruitment of circulating monocytes into the pancreatic stroma of KC mice remains unanswered. The increase of stromal macrophage numbers and inflammatory cytokines in the pancreas of mice harboring an oncogenic Kras, which is further enhanced in the obese state, suggests the existence of a positive reinforcement between mutated Kras and the obese microenvironment [67]. Signaling pathways responsive to and augmented by obesity, e.g., insulin/IGF-1 and neurotensin, can positively and synergistically reinforce signaling networks downstream of oncogenic Kras converging on YAP (yes-associated protein)/TAZ (transcriptional coactivator with PDZ-binding motif), transcriptional co-activators in the Hippo pathway, and critical nodes in PDAC [67,68,69].

**Figure 1:**
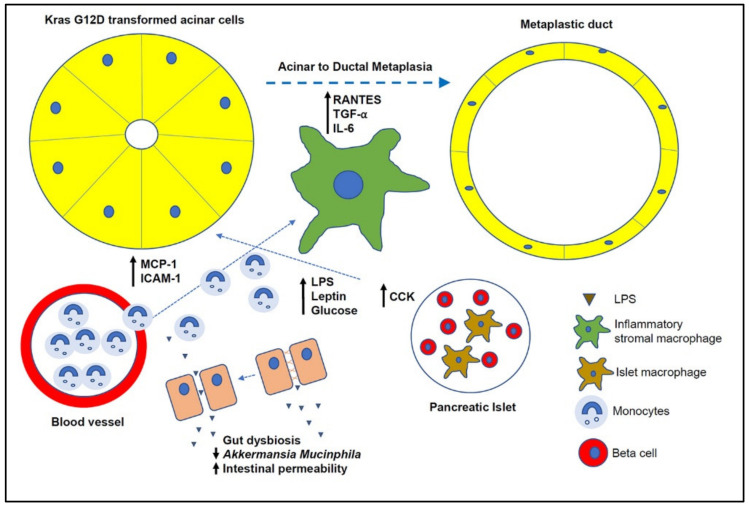
Role of pancreatic macrophages in obesity-accelerated early pancreatic neoplasia. During obesity, circulating monocytes are recruited into the pancreas where they differentiate into inflammatory macrophages driving acinar-to-ductal metaplasia. Several factors that recruit monocytes into the pancreas (e.g., MCP-1, ICAM-1) and polarize monocytes into inflammatory macrophages (e.g., LPS, leptin) are depicted here but others do exist. A communication between the endocrine and exocrine compartment in the pancreas driving this process is illustrated here as well. MCP-1: monocyte chemoattractant protein-1; ICAM-1: intercellular adhesion molecule 1; LPS: lipopolysaccharide; RANTES: regulated upon activation, normal T cell expressed and presumably secreted; TGF-α: transforming growth factor-alpha; IL-6: interleukin-6; CCK: cholecystokinin.

Similar to the effect of obesity on the adipose tissue, we postulate that during obesity circulating monocytes will be recruited to the pancreas where they differentiate into M1 polarized inflammatory macrophages, thereby resulting in the observed increased number of total pancreatic macrophages. These recruited inflammatory macrophages may drive an increased metaplastic program leading to enhanced ADM formation and PanIN/PDAC development (see above) (Figure 1).

During obesity, circulating Ly6C^+^ CCR2^high^ bone marrow-derived monocytes are recruited to the adipose tissue through a CCL2 (MCP-1)/CCR2-mediated mechanism [29,33,34]. It is conceivable that circulating monocytes are recruited to the pancreas through a similar mechanism in the obese condition. Human and mouse studies have documented that plasma levels of MCP-1 are elevated in obesity [70]. Although MCP-1 is ubiquitously expressed in various cell types by a wide variety of stimuli, adipocytes have been reported to be important sources of MCP-1 [70,71]. Our own studies have shown that MCP-1 levels are increased in the pancreas of KC mice and further substantially elevated in obese KC mice [65]. It has been reported that pancreatic acinar cells secret MCP-1 after stimulation with cholecystokinin (CCK) [72]. This is significant as a recent report described a novel and intriguing role of CCK in obesity-associated PDAC [73]. In this study, using KC mice crossed with leptin deficient *ob/ob* mice (KCO), obesity accelerates PDAC development through changes in the local pancreatic microenvironment, which is driven by increased pancreatic islet (β-cells)-secreted CCK acting locally on pancreatic acinar cells and accelerating ADM formation [73]. Intriguingly, elevated β-cell expression of CCK is mediated by obesity-associated activation of islet macrophages [73]. However, the exact signals underlying the activation of islet macrophages in the obese microenvironment are still unclear, although a role of hyperglycemia and glucotoxicity has been postulated [73,74]. It is plausible that islet-derived CCK in the obese state can also stimulate MCP-1 expression in pancreatic acinar cells, which recruits circulating monocytes into the pancreas, thereby further increasing inflammatory stromal macrophages and enhancing early pancreatic neoplasia.

Once recruited into the pancreas, monocytes can be polarized to inflammatory M1-like macrophages by a variety of signals that are elevated in the obese pancreatic microenvironment. Lipopolysaccharide (LPS, endotoxin), a component of the outer membrane of gram-negative bacteria, can potently polarize monocytes to inflammatory M1 macrophages in vitro and in vivo [75,76,77]. This effect is mediated by binding of LPS to its cognate receptor, toll-like receptor 4 (TLR4), in a complex with CD14 and MD2 on the cell surface of monocytes/macrophages [77]. LPS levels are elevated systemically and locally in tissues in diet-induced and genetic obesity in murine studies and this has been confirmed in humans as well [78,79,80,81,82]. Increased circulating levels of LPS, termed metabolic endotoxemia, are thought to contribute to the low-grade systemic inflammation seen in obesity and to the development of metabolic diseases, including type 2 diabetes mellitus. Animal studies have shown that metabolic endotoxemia is associated with an altered composition of the gut microbiota and an increased intestinal permeability [79,83,84]. There are several pathways and mechanisms by which obesity-induced gut dysbiosis can disrupt the intestinal barrier comprising of the intestinal epithelium, mucus layer, and factors released from the host innate and adaptive immune system, thereby increasing the intestinal permeability and translocation of LPS [84,85,86,87].

In this context, the intestinal symbiotic bacterium *Akkermansia muciniphila* has been shown to play a critical role in protecting the gut barrier function [88,89,90]. Human studies have provided evidence that the abundance of *Akkermansia muciniphila* is negatively correlated to obesity and other metabolic diseases [91,92,93]. In a randomized, double-blind, placebo-controlled study in overweight/obese and insulin-resistant human subjects, supplementation of *Akkermansia muciniphila* for three months slightly reduced body weight, improved insulin sensitivity, and reduced hyperinsulinemia and hypercholesterolemia together with a decrease in systemic inflammatory markers [94]. Our own studies have shown that administration of the anti-diabetic drug metformin to KC mice fed an obesogenic HFCD prevented weight gain, normalized hyperinsulinemia and hyperleptinemia, decreased pancreatic inflammation and attenuated PDAC development [95], which was associated with normalizing HFCD-induced gut dysbiosis [96]. In that study, oral administration of metformin to KC mice fed the HFCD decreased the genus *Clostridium sensu stricto* and substantially increased the abundance of *Akkermansia* [96]. It is conceivable that the improvement of the HFCD-induced gut dysbiosis in KC mice by metformin leads to a stabilization of the gut barrier and a decrease of circulating LPS, which consequently might reduce macrophage M1 polarization in the pancreas and attenuate ADM/PanIN formation. However, scientific evidence confirming this model is still lacking. Although we are not aware of any studies which directly demonstrated elevated LPS levels in the pancreas during obesity-associated PDAC development, other studies have suggested the importance of LPS for β-cell function in obese rats with deficiency in TLR4, the receptor for LPS [97]. In another report, administration of LPS to mice which express oncogenic Kras specifically in pancreatic acinar cells caused severe chronic pancreatitis and neoplastic PanIN lesions [98], further highlighting the importance of LPS for PDAC development, although the contribution of macrophages in LPS-treated mice was not addressed.

Besides LPS, other soluble factors elevated in obesity may also polarize monocytes recruited to the pancreas to inflammatory M1-like macrophages. Obesity is characterized by changes in adipokine secretion, including an increase in leptin, a 16-kDa peptide hormone, which is predominantly produced by the white adipose tissue [99,100,101,102]. As an anorexigenic hormone, leptin regulates body weight by suppressing appetite and stimulating energy expenditure through its central actions in the hypothalamus. In obesity, however, a central and peripheral leptin resistance develops leading to a disruption of the negative feedback loop between adipose tissue gain and satiety [102]. Animal models, in which either the leptin (*ob/ob* mouse) or leptin receptor (*db/db* mouse) genes are mutated, are characterized by profound obesity, hyperinsulinemia, and hyperglycemia [103,104]. Human studies have reported an association between elevated circulating leptin levels and PDAC risk [105,106]. In our murine studies, the acceleration of PDAC development in KC mice with DIO was associated with marked hyperleptinemia [65,66]. In another study, caloric restriction attenuated PanIN progression in the KC mouse model, which correlated with a reduction in circulating leptin levels [107]. Similarly, oral administration of metformin to KC mice fed an obesogenic diet led to reduced weight gain, normalization of elevated plasma leptin levels, and attenuation of PDAC development [95]. Besides its central anorexigenic effects, leptin also modulates a wide range of immune and inflammatory processes [108,109,110,111]. Leptin was shown to stimulate proliferation and activation of circulating monocytes and expression of pro-inflammatory cytokines, e.g., TNF-α and IL-6 [112,113,114]. Interestingly, LPS seems to synergize with leptin in producing IL-6 in macrophages and monocytes [115]. Conversely, macrophage phagocytosis was impaired in leptin-deficient *ob/ob* mice [116]. Similarly, in leptin receptor-deficient *db/db* mice, peritoneal and alveolar macrophages showed defective phagocytosis and were skewed towards M2 polarization [117,118]. Collectively, the available data convincingly suggest that leptin can stimulate pro-inflammatory cytokine production in macrophages, hence potentially contributing to the acceleration of PDAC development by acting on pancreatic macrophages to drive ADM and early PanIN formation/transition in the context of obesity with accompanied hyperleptinemia. However, a recent report demonstrating increased PDAC development in KC mice with genetic obesity (*ob/ob* mice) [73], which lack leptin production, questions the importance of leptin in obesity-associated PDAC. Yet, several differences exist between the genetic obesity and diet-induced obesity models, which typically are characterized by hyperleptinemia [74], and leptin still might play a critical role in human obesity-promoted PDAC.

Besides LPS and leptin, other factors that are elevated in the obese condition, such as saturated fatty acids and glucose, have been discussed as capable of polarizing macrophages towards an inflammatory phenotype but will not be discussed in this review. Instead, the reader is directed to several comprehensive reviews on this topic [38,76,119,120,121,122,123,124,125,126].

### 3.5. Intrapancreatic Adipose Tissue

**Figure 2:**
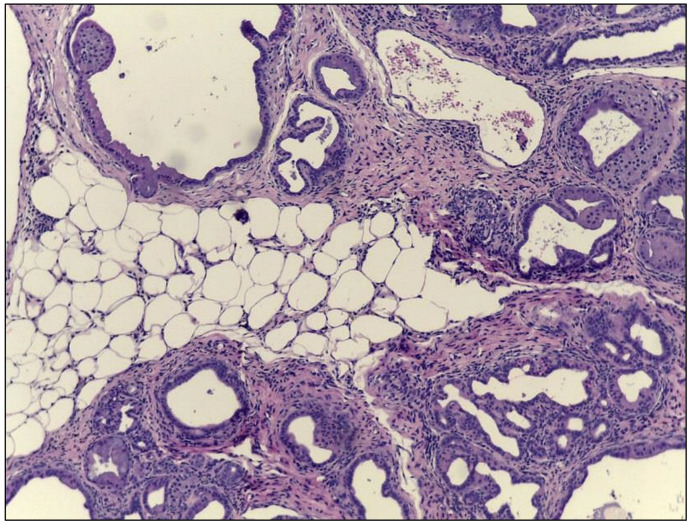
Intrapancreatic fat. H.E. (hematoxylin and eosin) image of the pancreas of a KC mouse with DIO (diet-induced obesity). The presence of adipocytes within the pancreatic parenchyma can be seen.

Although the existence of intrapancreatic fat has been known for several decades [127], our understanding of its origin and functional importance is very limited. Several studies have reported an increased fat deposition in the pancreas in human and murine obesity [127,128,129,130], and intrapancreatic fat has been suggested to contribute to the pathogenesis and severity of acute/chronic pancreatitis, T2DM, and PDAC [131,132,133,134]. Using computer tomography analyses the intrapancreatic fat volume was 32 and 68% greater in the overweight and obese groups, respectively, compared with lean subjects [130]. Patients that underwent bariatric surgery showed a decrease in intrapancreatic fat and an improvement of their glycemic status independent of changes in body weight and intraabdominal fat [135,136]. Interestingly, intrapancreatic fat was associated with elevated circulating levels of leptin and TNF-α independent of abdominal fat distribution in patients after acute pancreatitis [137]. Mice with oncogenic Kras expressed in acinar cells and deficient in pigment epithelium-derived factor (PEDF) showed a significant increase in intrapancreatic adipocytes, which was associated with a higher degree of ADMs, more frequently occurring cystic papillary neoplasms, and an increased incidence of invasive and metastatic PDAC [138]. In our own studies, we regularly observe adipocytes in the pancreas of KC mice with DIO (Figure 2).

However, whether the intrapancreatic adipocytes represent an intrusion of peripancreatic white adipocyte tissues or develop in the pancreas through differentiation of mesenchymal progenitor cells or a trans-differentiation program of, e.g., acinar cells, is currently unclear. Furthermore, whether intrapancreatic fat shows an inflammatory reaction in the obese state with increased macrophage recruitment and elevated cytokine production, similar to visceral adipose tissue, is unknown. Speculatively, pro-inflammatory macrophages and cytokines in inflamed intrapancreatic fat during obesity may directly promote early neoplastic development in the pancreas. However, this still needs to be investigated scientifically.

## 4. Conclusions

There is convincing evidence from human studies and mouse models that pancreatic macrophages play a critical role during PDAC development. Pancreatic macrophages, which comprise of a heterogenous population of mixed origin in the pancreas, at least contribute but may even be indispensable for exocrine and endocrine pancreatic pathology. Islet macrophages have been found to be important players in insulin resistance and β-cell dysfunction, while stromal inflammatory macrophages seem to drive early ADM formation and transition to PanINs. Although the role of macrophages in PDAC development in the context of obesity is largely unknown, it is conceivable, similar to obesity-associated AT inflammation, that circulating monocytes are recruited into the pancreas, where they are exposed to various factors that are increased in the obese tissue microenvironment, and differentiate into inflammatory macrophages, which then accelerate neoplastic development (Figure 1). Scientific confirmation is still needed but will provide the rationale for targeting macrophages to intercept early PDAC development.
